# The rise and fall of infectious disease in a warmer world

**DOI:** 10.12688/f1000research.8766.1

**Published:** 2016-08-19

**Authors:** Kevin D. Lafferty, Erin A. Mordecai

**Affiliations:** 1 Western Ecological Research Center, U.S. Geological Survey at Marine Science Institute, University of California, Santa Barbara, CA, 93106, USA; 2 Department of Biology, Stanford University, Stanford, CA, 94305, USA

**Keywords:** infectious disease, climate change, ecology

## Abstract

Now-outdated estimates proposed that climate change should have increased the number of people at risk of malaria, yet malaria and several other infectious diseases have declined. Although some diseases have increased as the climate has warmed, evidence for widespread climate-driven disease expansion has not materialized, despite increased research attention. Biological responses to warming depend on the non-linear relationships between physiological performance and temperature, called the thermal response curve. This leads performance to rise and fall with temperature. Under climate change, host species and their associated parasites face extinction if they cannot either thermoregulate or adapt by shifting phenology or geographic range. Climate change might also affect disease transmission through increases or decreases in host susceptibility and infective stage (and vector) production, longevity, and pathology. Many other factors drive disease transmission, especially economics, and some change in time along with temperature, making it hard to distinguish whether temperature drives disease or just correlates with disease drivers. Although it is difficult to predict how climate change will affect infectious disease, an ecological approach can help meet the challenge.

## Introduction

It is almost 2020, the year in which early efforts predicted that climate change would have increased the number of people at risk of malaria by 60% ^1^. As with many parasitic diseases, the malaria parasite and its mosquito vectors are most prevalent in the tropics and have annual peaks during or after warm seasons. Early climate models predicted that warming would expand parasite and mosquito vector ranges beyond the tropics. Furthermore, temperature change can stress hosts, and stress might make them more susceptible to infection or death. Finally, some diseases have emerged or increased ^2^ just as global greenhouse effects ^3^, urban heat islands ^4^, and devegetation ^5^ have increased temperatures over the last century, suggesting that this warmer world will be a sicker world ^6^. Concern for a sicker world led to increased research on climate change and infectious disease ^7^, and public opinion and funders took notice ^8^. As a result, we now better understand the complex linkages between climate and disease transmission.

Although the globe has warmed, many human infectious diseases (particularly those responsible for the most human suffering) have declined since 1999 ^9^, with some notable exceptions (for example, West Nile, Ebola, dengue, chikungunya, and Zika). For example, although warming has expanded highland malaria transmission in some places, transmission did not increase into western Europe and the United States ( Box 1) ^10^. Instead, after rising with population growth, mortalities declined by 40% in Africa between 2000 and 2015, and this was largely due to insecticide-treated bed nets and other interventions ^11^. The decline in malaria in a warming world shows how economic growth and interventions can mask climate effects. By contrast, other pathogens have expanded in recent years, particularly arboviruses such as West Nile, dengue, chikungunya, and Zika, pandemic influenza viruses, Ebola virus, and coronaviruses that cause severe acute respiratory syndrome (SARS) and Middle Eastern respiratory syndrome (MERS). The resurgence and spread of these diseases are thought to be linked to global change (for example, urbanization, land use change, climate change, and global travel), but direct links to climate change have not been established ^12^. In sum, many diseases are changing—some decreasing dramatically and others expanding and emerging—but the evidence linking these shifts to climate change is limited.

Box 1. **Malaria and climate**

The world’s most important parasitic disease is malaria ^13^. Climate affects the malaria parasite and the mosquitoes that vector it. However, there is a distinction between climate suitability for transmission and realized transmission because even when climate is suitable for malaria transmission, many additional factors interrupt transmission ^14^. In fact, economic development has trumped climate in determining the global malaria distribution, reducing malaria transmission in developed nations even as temperatures have become more permissive ^15^. Meanwhile, malaria resurged in Greece after health services had been cut following the 2008 recession (though some have blamed a warming climate as well) ^16^. In developing nations with limited vector control and healthcare infrastructure, climate can still hold sway. In particular, malaria has increased at high-altitudes in East Africa because of warming ^10^. Across a broader temperature range, climate impacts on malaria transmission are more subtle and include expanding the transmission season ^17^. At continental scales, some models project expansions in climate suitability for malaria ^18^, whereas other models, based on different curves, predict poleward (and altitudinal) shifts ^14, 19^ or decreases ^20^. These different predictions stem from different model assumptions. In particular, non-linearities in the performance curves for malaria and mosquitoes have suggested that optimal temperatures for transmission are much lower than previously thought ^21, 22^ and that in future warming scenarios much of Africa will be too warm for efficient transmission ^23^.

## Rise and fall

**Figure 1.**
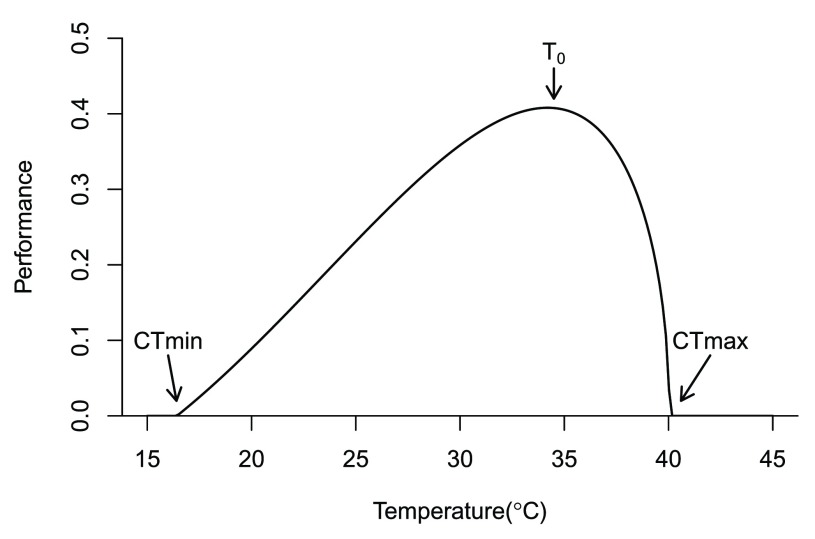
A thermal performance curve for a hypothetical ectotherm. All species, whether free-living or parasitic, rise and fall with temperature. Performance rises slowly from the critical thermal minimum (CTmin) to a thermal optimum (To), declining sharply to the critical thermal maximum (CTmax).

Biological responses to warming, including infectious diseases, depend on the non-linear relationships between performance and temperature. Temperature increases enzyme function ^24^, membrane permeability ^25^, and respiration rate ^26^ while decreasing molecular stability. These opposing temperature effects result in hump-shaped thermal reaction norms (or performance curves) that rise from a minimal temperature limit to a peak at the optimal temperature, followed by a sharp fall to a critical maximum temperature limit ^27^ ( Figure 1). When gene flow and acclimation are limited ^28^, laboratory organisms evolve to optimize performance by specializing on a fixed laboratory temperature ^29^, resulting in high and narrow performance curves. However, because field temperatures vary (daily, seasonally, annually, and decadally), especially with latitude, species either evolve low and broad thermal performance curves ^30^ or regulate toward an optimal temperature through endothermy, behavior, phenology, or migration ^31^. Such adaptations have established thermal niches that have bounded species distributions for millennia. But what happens if global temperatures increase faster than species’ thermal niches can adapt?

How any species, parasite or free-living, responds to increased global temperature depends on their current thermal response curve and their ability to thermoregulate, adapt (including shifts in phenology), and shift geographic ranges ^32^. For instance, species from high latitudes can tolerate considerable warming because they are adapted to variable temperatures ^33^. In contrast, most tropical species have narrow thermal response curves and limited thermoregulation ^33^. If such species are mobile, their geographic ranges should shift to higher latitudes and altitudes. In contrast, sedentary or island species might either adapt to warmer temperatures or go extinct. As parasite and host ranges shift, generalist parasites with broad host ranges should persist. By contrast, specialist parasites should be more sensitive because climate change could disrupt parasite transmission and reduce parasite burdens while also disrupting host populations. Host pathology could increase if infectious agents adapt faster to higher temperatures. This could be the case for long-lived, sedentary tropical hosts (like trees or corals) that have limited capacity to tolerate, adapt to, or move in response to temperature increases ^6^. Depending on the system, parasite transmission, pathology, and geographic range could decline, increase, or stay the same, making general predictions challenging ^34^.

Climate change might also affect disease transmission through host susceptibility, which depends, in part, on investment in defense. It is common to assume that warming will decrease immune function because cellular and humoral immune defenses are expensive to maintain ^35^ and can collapse under thermal stress. For instance, black abalone become more susceptible to rickettsia infection under variable temperatures and infected individuals die faster as mean temperature rises ^36^. On the other hand, warmer temperatures can increase immune response in fishes and amphibians ^37^, oak resistance to sudden oak death ^38^, and the immune response to cold virus in humans ^39^. Given these diverse responses, the immune response, like other performance measures, should follow a hump-shaped temperature response curve with decreased function at lower or higher than optimal temperatures, though too few data exist to generalize ^37^. Another way susceptibility can respond to climate change is if shifts in pathogen distributions expose naïve host populations to new diseases ^40^; a key example is occurring in the Ethiopian highlands, where recent malaria expansions have caused increased disease in previously unexposed populations ^41, 42^. Overall, climate change could affect host susceptibility, but the effect probably varies with temperature and other factors.

Temperature also affects parasite transmission through infective stage (and vector) production and longevity. For example, an influenza virion’s viability decreases with temperature so that flu transmission is lower during mild El Niño winters than in normal cold winters in California ^43^. In contrast, transmission stage production in ectothermic hosts should increase with temperature up to a thermal optimum; for example, nematode parasites have increased in warming Arctic areas where larval development rates have increased ^44, 45^. Increases in parasite and host death rates can counteract this temperature effect on parasite production. For instance, snails shed more amphibian-seeking trematode larvae under warmer temperatures ^46^, and one might expect that parasite intensity would increase. However, trematode intensity is halved in a 3 **°**C warming experiment because of an increase in parasite and host death rate ^47^. This dual effect of production and death on transmission helps explain why some trematode infections increase with warmer water ^48^, whereas other species do better at cold temperatures ^49^. Likewise, although field observations initially suggested that swallow parasites would increase with global warming, experimentally heating nests above ambient temperature killed parasites, leading to a sharp decline in parasitism ^50^. Similarly, increasing temperature hastens *Plasmodium* development but, above a certain temperature, transmission declines because of increased mosquito death, leading to hump-shaped temperature-transmission curves ^51^.

The shape and range of a thermal response curve ( Figure 1) determine where temperature suitability for an infectious disease will increase or decrease with climate change, with differences in model curves altering model predictions ( Box 1). Potential poleward shifts in suitability for tropical diseases predicted from the thermal performance curve can cause alarm in countries at high latitudes ^52^. Fortunately, many high-latitude countries are able to mitigate such risks because of higher economic development and healthcare infrastructure ^53^.

Host pathology should often follow the parasite thermal performance curve ^54^. For instance, the fungal pathogens responsible for amphibian declines have a cool optimal temperature ^55^ and are expected to cause fewer problems as climate warms ^56^. However, host death rates tend to increase with temperature, as seen for infected corals ^57, 58^, sea stars ^59, 60^, and abalone ^61, 62^ that have suffered catastrophic mass mortalities. Because temperature-induced host death kills parasites, it can, in the long term, drive a host-specialist parasite to extinction ^36, 63^. For this reason, the most pathogenic parasites have a hard time persisting without a tolerant reservoir host.

## Other drivers

Many factors drive disease transmission, including some that have changed in time along with climate. A key factor is host density (including vectors and reservoir hosts), which should increase the force of infection ^64^. For instance, although marine disease reports have increased over time ^65^, and some, like coral diseases, might be due to increased temperature, most disease reports parallel changes in host abundance (due to either increased disease transmission or increased detection). In particular, the one group for which disease reports declined over time was commercially valuable fishes, which have suffered global stock collapses ^66^. For human, crop, and livestock diseases, economic development is another key driver ^67, 68^. Most notably, it is clear from historical data that malaria does not occupy its full ecological niche, due to its elimination from wealthier nations ^69^, leading to a decline in malaria’s distribution as the earth has warmed ^15, 34^. Economic growth drives health interventions such as vector control, increased sanitation, deforestation, and shifts to urban living with limited wildlife contact, all of which can reduce disease emergence and spread ^70^. However, economic growth also drives global travel and trade, which can unintentionally spread pathogens. Contact networks for directly transmitted diseases are now global due to air travel and trade routes that continue to introduce invasive species, which, on average, bring with them two parasitic species per host species ^42^. These novel parasites meet naïve hosts with unpredictable outcomes. Because host density and economics change over time, it can be hard to separate climate change effects from other changes. Current examples are emerging and resurging dengue, chikungunya, and Zika viruses. These viruses benefitted from vector expansion and invasion, lapsed vector control, global tire trade, global human movement, urbanization and unplanned development, deforestation, high human densities, and poverty ^71^. Despite these drivers, climate change captures the headlines, such as “ *In Zika Epidemic, a Warning on Climate Change*” ^72^.

Many emerging and resurging diseases in wildlife (for example, chytridiomycosis, and white nose syndrome) and in humans (for example, dengue, Zika, chikungunya, and diarrheal diseases) are increasing in prevalence and geographic range over time as climate changes, constituting the main evidence to link a warmer world with a sicker world ^7, 73^. However, other drivers can correlate with temperature changes, making it difficult to separate a temperature effect from a spurious correlation. In particular, disease events during warm years are not sufficient evidence for a climate change effect because diseases emerge each year for various reasons. For instance, seven out of nine historical yellow fever epidemics occurred in different cities during the 1878–79 El Niño ^74^, but this strong El Niño effect disappears after considering longer time series ^34^. Because the recent Brazilian Zika epidemic occurred during an El Niño, similar claims have been made for Zika virus ^75^ even though Zika had previously spread throughout the South Pacific without fanfare ^76^. Although increases in new human diseases and new locations for old diseases are alarming, some may be due to species introductions or increased surveillance and reporting but have no clear link to climate ^2^. Sometimes a causal link between temperature and disease is not due to thermal physiology or climate change. In addition to direct physiological effects, temperature can indirectly affect disease dynamics. A temperature association can occur if warming drives changes in abundance or movement that affect transmission. For example, warming increases disease in monarch butterflies because a milder climate removes the need to migrate, which benefits protozoan parasites otherwise lost when infected hosts die during migration ^77^. In other cases, a temperature effect is not a climate change effect. For example, deforestation creates warmer microclimates, which, in turn, can increase local malaria transmission ^78^. Regardless, the difficulty in establishing a link between climate change and disease does not mean no link exists, just that observed links have alternative explanations that might be more or less likely. For these reasons, temperature-disease correlations best indicate a climate change effect if they persist after removing the temporal trend and have experimental support.

When climate does associate with an increase in a disease in a particular location, it is important not to overgeneralize. Sometimes effects vary among hosts, such as the observation that parasites increased in some but not all European bird species over 5- to 15-year time intervals, with a positive association between changes in temperature and changes in parasitism ^79^. Results can also be inconsistent among similar parasite species. For instance, a 30-year study found that one rabbit nematode species increased along with increases in temperatures but that a second did not change ^80^. Similarly, while temperature increased at Finnish fish farms, two pathogens increased and two declined ^81^. Finally, an increase in one location might be paired with a decrease at another location ^23^. Although general explanations are attractive, we should expect winners and losers as climate changes.

## Conclusion

Although it is difficult to test how climate affects infectious disease burdens in humans, livestock, and wildlife, the implications for human wellbeing make it imperative that we meet the challenge. Fortunately, climatologists are making headway defining climate change, and their efforts could lead to new insights into potential disease drivers like disproportionate increases in nighttime or winter temperatures, reduced temperature variation, increased extreme event intensity and frequency, and changes to precipitation. Armed with better climate information, ecologists can use experiments, mathematical and statistical modeling, and observational work to understand and predict how infectious disease responds to climate change ^82^. Basic information on thermal physiology is lacking, but various efforts are underway to better describe infectious agent and vector thermal niches by describing their thermal performance curves, testing for local adaptation, and measuring thermoregulation. Once we understand thermal physiology better, a greater appreciation for the economic and environmental factors driving infectious diseases will make it easier to evaluate climate change effects in relation to parallel changes such as land conversion, urbanization, species assemblages, host movement, and demography ^83^. At that point, we can predict which diseases are most likely to emerge where, so that public health agencies can best direct limited disease control resources ^84^, rather than wondering whether a warmer world will be a sicker world.
